# Depression, anxiety, anger, and loneliness in older adults: comparing residential contexts and examining the role of loneliness

**DOI:** 10.1186/s12877-026-07621-5

**Published:** 2026-05-15

**Authors:** Elena Ruiz-Sancho, Miguel Ángel Pérez-Nieto, Francisco Sánchez-Escamilla, Lucia Torices, Patricia Cañada, Enrique Rubio, Andrea Aguirre, Marta Redondo-Delgado

**Affiliations:** 1 Facultad HM de Ciencias de la Salud, Universidad Camilo José Cela, c/ Castillo de Alarcón, 49. 28692 Villanueva de la Cañada, Madrid, Spain; 2 Instituto de Investigación Sanitaria HM Hospitales, Madrid, 28015 Spain; 3 Institute of Psychology of Emotion and Health, IPES, Madrid, Spain

**Keywords:** Anger, Anxiety, Depression, Explanatory models, Institutionalization, Loneliness, Older adults

## Abstract

**Background:**

Population ageing increases the need to understand psychosocial determinants of emotional well-being in later life. Loneliness is a key predictor of depression and anxiety in older adults, yet other emotions such as anger—less frequently investigated in gerontological research—may also contribute to distress when social needs are unmet. Although residential context has been linked to emotional health, the extent to which loneliness helps explain this relationship remains unclear. This study aimed to (1) explore differences in depression, anxiety, anger, and loneliness between institutionalized and community-dwelling older adults, and (2) examine the role of loneliness in the relationship between residential status, age, and emotional outcomes.

**Method:**

A cross-sectional study was conducted with 190 participants aged 60 years and older (M = 79.0, SD = 9.15). Participants were recruited from nursing homes, senior centers, and community settings in Spain through institutional collaboration and snowball sampling. Measures included the Geriatric Depression Scale (GDS-15), Geriatric Anxiety Inventory (GAI), State–Trait Anger Expression Inventory (STAXI-2, trait anger scale), and the UCLA Loneliness Scale (10-item version). Data analyses comprised ANCOVAs controlling for age and three explanatory models estimated with Jamovi (GLM Mediation, version 2.6.24).

**Results:**

Institutionalized participants reported significantly higher depression scores, with mean scores at the commonly used GDS-15 screening threshold. They also showed a tendency toward greater anger and loneliness, while no group differences were found in anxiety. The models indicated that loneliness was a consistent and robust factor associated with depression, anxiety, and anger. However, neither age nor residential status showed significant indirect effects through loneliness. The total effect of residential status on depression was significant, whereas no significant direct or indirect effects of residential status or age were observed for anxiety and anger. The models explained 27.4% of the variance in depression, 23.4% in anxiety, and 17.7% in anger.

**Conclusions:**

Loneliness emerged as the factor most consistently associated with emotional distress in older adults. These findings underscore the importance of designing public policies and psychosocial interventions focused on reducing unwanted loneliness and strengthening social connectedness in both institutional and community settings.

## Background

Population ageing is a global reality undergoing progressive growth. According to recent estimates, more than 10% of the world’s population was aged 65 years or older in 2024, and this proportion is expected to increase to nearly 22% by 2050, reaching over 1.7 billion older adults worldwide [1]. This demographic shift poses significant challenges for health systems, social services, and public policy, while also demanding renewed attention to the factors that shape the emotional well-being of older people in diverse living contexts.

From a psychosocial vulnerability perspective, ageing does not constitute a homogeneous or linear stage. Instead, its impact is strongly mediated by variables such as health status, functionality, economic conditions, residential environment, and perceived social support [2, 3]. Rodríguez Martín’s [4] crisis model of ageing identifies three critical dimensions of vulnerability: identity crisis, linked to the loss of significant roles; autonomy crisis, related to functional dependence; and belonging crisis, associated with changes or disruptions in social and affective ties. Although close and meaningful relationships may become more salient in later life, reductions in broader social networks or losses of significant relationships may still contribute to emotional vulnerability.

Depression and anxiety are two of the most common emotional disorders among older adults. Both are associated with multiple adverse consequences, including functional decline, greater physical morbidity, cognitive deterioration, increased health service utilization, and a marked reduction in quality of life [5, 6]. Detection is often difficult, as symptoms are frequently mistaken for normal ageing processes, especially among institutionalized individuals, where care models tend to prioritize physical rather than emotional needs [7]. In addition, less recognized emotions such as anger may arise as responses to frustration, dependency, invisibility, or loss of control over one’s environment. At the same time, anger may be more difficult to identify or may be expressed less openly in later life, sometimes emerging in more indirect forms. This may partly explain why anger has received less attention than depression or anxiety in research on older adults, despite its potential relevance to emotional distress. Although less frequently studied, anger has been linked to heightened anxiety in older populations and may represent an indirect pathway toward emotional deterioration if not adequately managed [8].

A key variable that cuts across these emotional states is the living environment. Several studies have shown significant differences between older adults living in their own homes and those residing in institutions, both in terms of physical health and emotional well-being [9, 10]. Life in residential care facilities can entail a complete reorganization of living space, loss of autonomy, diminished exposure to familiar emotional stimuli, and rupture of family ties. These conditions may foster persistent feelings of alienation and isolation, particularly when opportunities for participation or meaningful relationships within the new setting are lacking [11]. Conversely, older adults who remain at home often benefit from maintaining daily routines and exercising greater decision-making autonomy [12, 13], although they may still experience social isolation if lacking active support networks. Other studies emphasize that residential care does not uniformly produce adverse effects. In some institutional contexts where group activities, personalized care, and opportunities for social engagement are promoted, older adults may develop new forms of belonging and report reduced negative affect [14, 15]. However, such cases remain the exception and are heavily influenced by variables such as facility type, quality of care, degree of autonomy allowed, and continuity of external ties [16]. At the same time, residential status should be interpreted with caution. The distinction between institutionalized and community-dwelling older adults is useful for descriptive and comparative purposes, but it may oversimplify heterogeneous living situations within both groups. In addition, institutionalization is often preceded by cumulative vulnerabilities, including functional decline, health problems, loss of close relationships, and reduced social support. Therefore, differences associated with residential status should not be understood as resulting from living context alone, but rather as potentially reflecting broader psychosocial and care-related processes. In this context, loneliness emerges as a central variable in understanding the emotional state of older adults in both institutional and community settings.

Loneliness has been consistently identified in the literature as one of the strongest predictors of emotional decline in old age [17, 18]. Far from being an exclusively objective phenomenon—merely linked to being alone—it is defined as the subjective perception of a discrepancy between desired and available social relationships. This disconnection may manifest across three dimensions: emotional loneliness (absence of intimate relationships), social loneliness (lack of belonging to networks or communities), and existential loneliness (feelings of emptiness or lack of life meaning) [19]. Recent studies report that up to 70% of institutionalized older adults experience some degree of loneliness, with particularly high prevalence of emotional and social loneliness [16]. The loss of meaningful relationships, disruptions in social support networks, and the inability to actively participate in social life foster the chronicity of this experience, which is rarely openly expressed by residents [20, 21]. These findings are particularly relevant given that loneliness has been consistently associated with depression, anxiety, cognitive impairment, sleep disturbances, and increased mortality [22, 23].

Recent research suggests that loneliness may not only act as an independent risk factor but may also help explain the relationship between other variables—such as age or type of residence—and the emergence of emotional symptoms. In other words, rather than advanced age or institutionalization per se, it may be the subjective experience of loneliness that best explains emotional decline and depression in later life [24]. However, such models remain scarce and seldom include the joint analysis of other relevant variables such as anger or anxiety.

Considering these aspects, the present study seeks to contribute to the understanding of emotional distress in old age through two main objectives: (1) to explore differences in anxiety, depression, anger, and loneliness between institutionalized and community-dwelling older adults, and (2) to examine the role of loneliness in the relationship between residential status, age, and emotional symptoms (depression, anxiety, and anger). This approach aims to detect patterns of emotional vulnerability and to inform psychosocial interventions that prioritize strengthening affective ties, fostering a sense of belonging, and promoting emotional well-being among diverse segments of the older population.

## Methods

### Participants

A total of 198 individuals participated in the study between July 2023 and January 2024. Participants were recruited from nursing homes and senior centers in various regions of Spain, as well as through a snowball sampling method. Individuals were classified as institutionalized if they resided permanently in nursing homes or long-term care facilities, while non-institutionalized participants were those living in their own homes. All participants provided written informed consent prior to enrollment in the study.

The final sample consisted of 190 participants after excluding individuals who made more than two errors on the Short Portable Mental State Questionnaire (SPMSQ; [25]; Spanish adaptation [26]), which was used as a screening tool for mild cognitive impairment. The inclusion criterion was being over 60 years of age, following the World Health Organization’s definition of older adulthood [27]. The exclusion criterion was the presence of cognitive impairment. Table 1 presents the demographic characteristics of the total sample, as well as those of the institutionalized and non-institutionalized groups.

**Table 1:** Characteristics of the total sample and of the institutionalized and non-institutionalized groups

	Total sample *N* = 190		Non-institutionalized *N* = 112		Institutionalized *N* = 78	
	Mean (SD) Range	*N* (%)	Mean (SD) Range	*N* (%)	Mean (SD) Range	*N* (%)
Age	79.0 (9.15) 61–102		75.3 (8.02) 61–101		84.3 (8.01) 65–102	
Sex						
Women		132(69.5)		84(75.0)		48 (61.54)
Men		58(30.5)		28(25.7)		30 (38.46)
Employment status						
Currently working		2(1.1)		2(1.78)		0(0)
Homemaker		19 (10.0)		19(16.96)		0(0)
On sick leave		2 (1.1)		2(1.78)		0(0)
Retired		165 (86.8)		87(77.67)		78(100)
Marital status						
Single		27 (14.2)		21(18.75)		6(7.69)
Married/ Partnered		82 (43.2)		56(50.0)		26(33.33)
Separated/ Divorced		14 (7.4)		8(7.14)		6(7.69)
Widowed		67 (35.3)		27(24.10)		40(51.28)

*SD* Standard Deviation

The sample was composed predominantly of women. Most participants were retired. Regarding marital status, married or widowed individuals were the most frequent. To verify baseline equivalence between groups, differences in sex and age were examined. The chi-square test showed no significant differences in sex distribution, χ²(1, *N* = 190) = 2.84, *p* = .092, whereas the independent samples t-test indicated significant differences in age, t(196) = − 7.66, *p* < .001.

### Instruments

#### Ad hoc questionnaire for sociodemographic variables

Sociodemographic data were collected through an ad hoc questionnaire that included the following variables: sex, age, marital status, and employment status.

#### Yesavage Geriatric Depression Scale (GDS-15)

The Geriatric Depression Scale was developed to assess depression in older adults without overestimating somatic or neurovegetative symptoms. A widely used shortened 15-item version was later introduced [28]. Items follow a Yes/No response format, with Items 1, 5, 7, 11, and 13 being reverse scored to align with the direction of the remaining items. In this study, “Yes” responses were coded as 1 and “No” responses as 0, such that higher scores indicate greater depressive symptomatology. The instrument shows good sensitivity and specificity values, making it a widely recommended tool for general screening of depression in geriatric populations [29, 30]. In the present sample, internal consistency was acceptable (Cronbach’s α = 0.797).

#### Geriatric Anxiety Inventory (GAI)

The GAI is a 20-item questionnaire designed to measure anxiety in older adults [31]. Items are rated dichotomously (1 = “agree,” 2 = “disagree”). For the present study, given that all items are positively worded, responses were recoded as 1 = “agree” and 0 = “disagree,” with higher scores reflecting greater anxiety levels. Prior studies have confirmed the adequate psychometric properties of this instrument [31, 32]. In the present sample, internal consistency was excellent (Cronbach’s α = 0.911).

#### State–Trait Anger Expression Inventory (STAXI-2)

The STAXI-2 is designed to assess different facets of anger. The Spanish version [33] includes 49 items structured across six scales. For this study, the trait scale (10 items) was selected, which evaluates how participants typically perceive situations as annoying or frustrating and their tendency to respond with anger. This scale includes items referring to the general tendency to feel irritated, treated unfairly, or provoked, and therefore captures dispositional aspects of anger rather than overt aggressive behavior. Although other scales may yield additional insights, the general trait of anger was prioritized for screening purposes. Responses are scored on a 4-point scale ranging from 1 = “almost never” to 4 = “almost always,” based on how participants generally feel. The instrument demonstrates satisfactory test–retest reliability and Cronbach’s alpha coefficients [33]. Notably, no instrument specifically designed to measure anger in older adults has been identified; therefore, the STAXI-2 was selected given its prior application in studies with this population [34, 35]. In the present sample, internal consistency was good (Cronbach’s α = 0.843).

#### UCLA loneliness scale

The UCLA Loneliness Scale is one of the most widely used instruments for measuring loneliness. While the original version consists of 20 items, several shortened versions have been developed. The 10-item version by Russell [36] was used in this study. Items are rated from 1 = “I often feel that way” to 4 = “I never feel that way.” To ensure consistency in interpretation, items were reverse coded so that 1 = “I never feel that way” and 4 = “I often feel that way,” with higher scores reflecting greater loneliness. This scale has demonstrated adequate psychometric sensitivity for measuring loneliness in older adults and has been validated in this population [37]. In the present sample, internal consistency was good (Cronbach’s α = 0.857).

### Procedure

Data collection was conducted between July 2023 and January 2024. Participants were recruited through two complementary strategies: (a) via eight residential and day care centers located in different Spanish cities, and (b) through snowball sampling. For institutional settings, directors were contacted in advance by email to request their collaboration in the study. Participants were classified as institutionalized if they resided permanently in nursing homes or long-term care facilities, and as non-institutionalized if they lived in their own homes. Because recruitment combined institutional collaboration and snowball sampling, the sample should be interpreted as a convenience sample rather than a representative population-based sample.

In all cases, participants were informed about the aims of the research, assured of the confidentiality of their responses, and reminded of their right to participate voluntarily. Written informed consent was obtained before completing the instruments. Questionnaires were administered individually, either in person or online depending on the context, with a psychologist available to provide support and clarify any questions during the process. The study was approved by the Ethics Committee of Universidad Camilo José Cela (14_23_SPM, CEI-UCJC). All procedures performed in this study involving human participants were in accordance with relevant guidelines and regulations, including the Declaration of Helsinki.

### Design and data analysis

A quantitative, cross-sectional, ex post facto design was employed. First, descriptive analyses were conducted for all variables. To verify baseline equivalence between groups (institutionalized vs. non-institutionalized), Student’s *t*-tests and chi-square tests were applied.

To address the first objective, analyses of covariance (ANCOVAs) were performed to compare groups on depression, anxiety, anger, and loneliness while controlling for age as a covariate. The assumption of homogeneity of regression slopes was explored by testing the interaction between age and residential status in each model. No clear violations were observed, although the interaction term in the depression model was close to the conventional significance threshold. To examine the second objective, three models were estimated using the General Linear Model (GLM) Mediation module in Jamovi (version 2.6.24), considering depression, anxiety, and anger as dependent variables. In each model, age and residential status were included as independent variables, and loneliness as the explanatory variable. Residential status was coded as 0 = community-dwelling and 1 = institutionalized. Effect sizes were estimated using standardized coefficients (β), and the significance of indirect effects was evaluated through 95% confidence intervals obtained via bootstrapping with 10,000 resamples and BCa correction.

Regression assumptions were examined. Collinearity analyses indicated VIF values of approximately 1.3, ruling out multicollinearity concerns. Plots of residuals against fitted values and predictors revealed an approximately linear distribution with no relevant curvilinear patterns. However, normality tests (Shapiro–Wilk, *p* < .001) and Q-Q plots indicated deviations from normality, as expected given the discrete nature of the variables. To mitigate this limitation and ensure the robustness of results, bootstrap estimation was employed. Although no a priori power analysis was conducted, the final sample size (*N* = 190) provided sufficient power (> 0.80) to detect small-to-moderate effects (f² ≥ 0.06) in the ANCOVA and model-based analyses, according to conventional benchmarks [38].

## Results

To address the first objective, Table 2 presents the descriptive analyses and group comparisons between institutionalized and non-institutionalized older adults in depression, anxiety, anger, and loneliness.

**Table 2:** Means, standard deviations, and ANCOVA results for depression, anxiety, anger, and loneliness among institutionalized and non-institutionalized older adults, controlling for age

Variable	Total sample M (SD)	Non-institutionalized M (SD)	Institutionalized M (SD)	F (1, 187)	*p*	η²*p*
Depression (GDS)	3.67 (3.23)	2.98 (2.95)	4.51 (3.37)	6.42	0.012	0.033
Anxiety (GAI)	6.50 (5.62)	6.19 (5.89)	6.88 (5.27)	0.34	0.559	0.002
Anger (STAXI)	18.9 (5.86)	18.2 (5.41)	20.0 (6.29)	3.99	0.047	0.020
Loneliness (UCLA)	18.5 (6.48)	17.9 (6.20)	19.4 (6.78)	2.95	0.088	0.015

*M* mean, *SD *standard deviation, *F *Fisher’s statistic in ANCOVA; values in parentheses indicate the effect and error degrees of freedom; *p* = significance level; partial η² = partial eta squared (effect size)

**Fig. 1:**
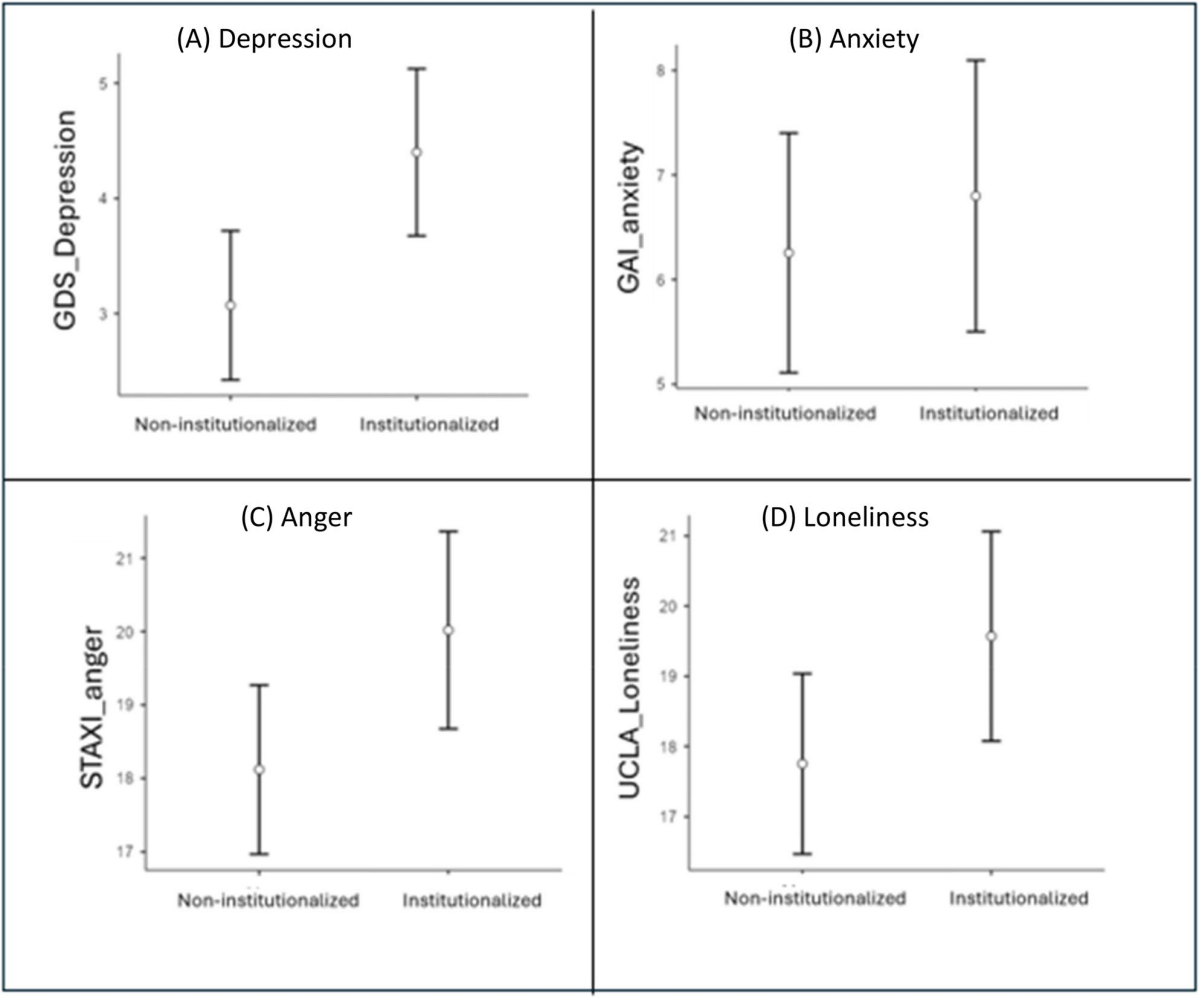
Adjusted means by residential status for (**A**) depression, (**B**) anxiety, (**C**) anger, and (**D**) loneliness. Error bars represent standard errors

The analyses showed that institutionalized participants reported higher levels of depression and, to a lesser extent, anger compared to their non-institutionalized counterparts. Effect sizes were small overall, although the effect for depression was larger than those observed for the other emotional variables. No differences were observed for anxiety, and for loneliness only a trend toward higher scores was found in the institutionalized group, also with a small effect size. To facilitate interpretation, Fig. 1 display the adjusted means of each variable by residential status, providing a clearer visualization of the magnitude and direction of group differences.

Overall, institutionalized participants tended to report higher scores than community-dwelling participants, particularly for depression and anger, where group differences were more evident. By contrast, anxiety scores were similar between groups, and loneliness showed only a non-significant tendency toward higher values in the institutionalized group.

To address the second objective, Table 3 summarizes the results of the models for depression, anxiety, and anger, including the direct, indirect, and total effects of age and residential status, with loneliness as the explanatory variable. The table reports standardized coefficients (β), 95% confidence intervals obtained through the bootstrap procedure, as well as standard errors and statistical significance values.

**Table 3:** Direct, indirect, and total effects of the models with loneliness as the explanatory variable

Model	Type	Effect	Estimate	SE	95% CI (a)		β	z	*p*
					Lower	Upper			
Depression	Indirect								
	Age ⇒ Loneliness (UCLA) ⇒ Depression (GDS)		-0.00528	0.0137	-0.0344	0.0208	-0.0160	-0.386	0.699
	Residential status ⇒ Loneliness (UCLA) ⇒ Depression (GDS)		0.31821	0.2591	-0.1814	0.9650	0.0514	1.228	0.219
	Component								
	Age ⇒ Loneliness (UCLA)		-0.02309	0.0597	-0.1426	0.0925	-0.0328	-0.387	0.699
	Loneliness (UCLA) ⇒ Depression (GDS)		0.22852	0.0297	0.1434	0.3043	0.4875	7.683	< 0.001
	Residential status ⇒ Loneliness (UCLA)		1.39252	1.1194	-0.9382	3.8042	0.1055	1.244	0.214
	Direct								
	Age ⇒ Depression (GDS)		0.02087	0.0240	-0.0281	0.0653	0.0632	0.871	0.384
	Residential status ⇒ Depression (GDS)		0.71741	0.4511	-0.1551	1.5682	0.1159	1.590	0.112
	Total								
	Age ⇒ Depression (GDS)		0.01544	0.0276	-0.0394	0.0668	0.0467	0.559	0.576
	Residential status ⇒ Depression (GDS)		1.05627	0.5166	0.0423	2.1725	0.1707	2.045	0.041
Anxiety	Indirect								
	Age ⇒ Loneliness (UCLA) ⇒ Anxiety (GAI)		-0.00345	0.0250	-0.0571	0.0459	-0.00560	-0.138	0.890
	Residential status ⇒ Loneliness (UCLA) ⇒ Anxiety (GAI)		0.62348	0.4658	-0.2842	1.6959	0.05525	1.338	0.181
	Component								
	Age ⇒ Loneliness (UCLA)		-0.00829	0.0601	-0.1318	0.1087	-0.01156	-0.138	0.890
	Loneliness (UCLA) ⇒ Anxiety (GAI)		0.41545	0.0558	0.2917	0.5330	0.48431	7.442	< 0.001
	Residential status ⇒ Loneliness (UCLA)		1.50074	1.1030	-0.7636	3.8217	0.11407	1.361	0.174
	Direct								
	Age ⇒ Anxiety (GAI)		0.01767	0.0454	-0.0679	0.1067	0.02872	0.389	0.697
	Residential status ⇒ Anxiety (GAI)		-0.25059	0.8372	-1.8410	1.2742	-0.02221	-0.299	0.765
	Total								
	Age ⇒ Anxiety (GAI)		0.01426	0.0518	-0.0833	0.1101	0.02319	0.275	0.783
	Residential status ⇒ Anxiety (GAI)		0.36490	0.9492	-1.3685	2.1633	0.03240	0.384	0.701
Anger	Indirect								
	Age ⇒ Loneliness (UCLA) ⇒ Anger (STAXI)		-0.00957	0.0210	-0.0580	0.0287	-0.01510	-0.4569	0.648
	Residential status ⇒ Loneliness (UCLA) ⇒ Anger (STAXI)		0.56229	0.3992	-0.1517	1.4671	0.04775	1.4085	0.159
	Component								
	Age ⇒ Loneliness (UCLA)		-0.02685	0.0586	-0.1480	0.0830	-0.03791	-0.4582	0.647
	Loneliness (UCLA) ⇒ Anger (STAXI)		0.35649	0.0594	0.2170	0.4951	0.39832	6.0013	< 0.001
	Residential status ⇒ Loneliness (UCLA)		1.57729	1.0885	-0.6394	3.8812	0.11988	1.4490	0.147
	Direct								
	Residential status ⇒ Anger (STAXI)		1.21550	0.8939	-0.5064	2.9829	0.10322	1.3598	0.174
	Age ⇒ Anger (STAXI)		-0.00436	0.0479	-0.1067	0.0878	-0.00688	-0.0911	0.927
	Total								
	Residential status ⇒ Anger (STAXI)		1.72196	0.9711	-0.0650	3.5805	0.14619	1.7732	0.076
	Age ⇒ Anger (STAXI)		-0.01358	0.0524	-0.1030	0.0844	-0.02138	-0.2593	0.795

95% confidence intervals (CIs) were calculated using bias-corrected (BC) bootstrap with 10,000 resamples. Standardized effects are reported as β coefficients. The number of cases included varied across models due to missing data in the variables entered in each analysis: depression model, *n* = 182; anxiety model, *n* = 183; anger model, *n* = 189

The models revealed a consistent pattern across the three dependent variables. In all cases, loneliness was significantly and positively associated with higher levels of depression, anxiety, and anger, confirming its role as a consistent correlate of psychological distress in old age. The interpretation of effects followed the criterion of bootstrap-derived confidence intervals, with significance determined only when the interval did not include zero [39]. According to this criterion, neither age nor residential status was significantly related to loneliness, nor did they exert significant indirect effects on the dependent variables through it. For depression, however, the total effect of residential status on symptoms was significant, indicating an association beyond the role of loneliness. However, the direct effect was not statistically significant. In contrast, for anxiety and anger, no relevant direct or indirect effects of residential status or age were observed.

In terms of explained variance, the depression model accounted for 27.4% of the variance, whereas the anxiety and anger models explained 23.4% and 17.7%, respectively, indicating that loneliness makes a substantial contribution to the understanding of these emotional variables.

**Fig. 2:**
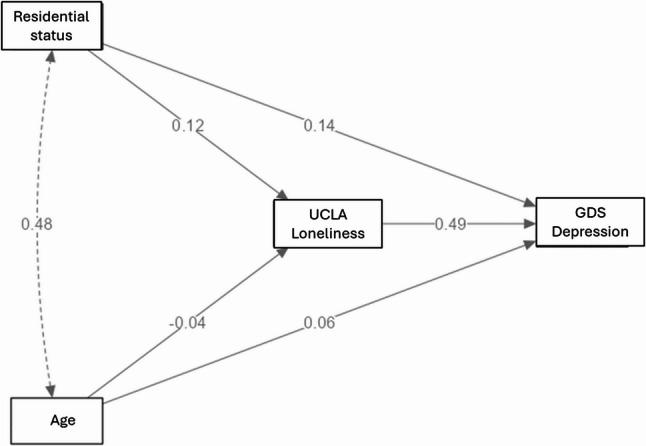
Explanatory model for depression (GDS), with loneliness (UCLA) as the explanatory variable. Standardized coefficients (β) are shown for each path

**Fig. 3:**
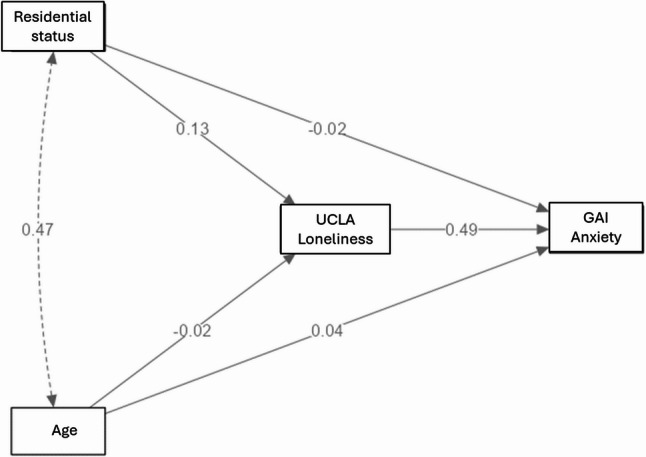
Explanatory model for anxiety (GAI), with loneliness (UCLA) as the explanatory variable. Standardized coefficients (β) are shown for each path

**Fig. 4:**
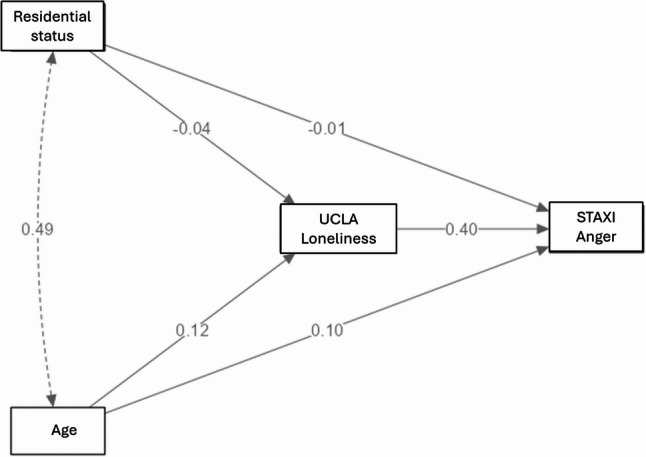
Explanatory model for anger (STAXI), with loneliness (UCLA) as the explanatory variable. Standardized coefficients (β) are shown for each path

To facilitate visual interpretation of these results, Figs. 2, 3 and 4 present the explanatory diagrams for the depression, anxiety, and anger models, respectively, reporting standardized coefficients (β) for each relationship among variables.

Across all three models, loneliness showed direct and positive associations with levels of depression, anxiety, and anger, highlighting its consistent association with psychological distress in old age. The effect was strongest in the depression and anxiety models (β = 0.49), followed by anger (β = 0.40)

## Discussion

The findings of this study provide evidence regarding the role of loneliness and residential context in the emotional well-being of older adults. At a descriptive level, the overall sample reported moderate levels of depression, anxiety, anger, and loneliness, consistent with data reported in previous studies [18, 40–42]. These levels highlight the psychological vulnerability of later life, reinforcing the need to consider emotional distress as a central aspect of aging [43].

With respect to the first objective—exploring differences in anxiety, depression, anger, and loneliness between institutionalized and community-dwelling older adults—the former group presented significantly higher scores for depression, as well as a tendency toward higher levels of anger and loneliness. Specifically, among institutionalized participants, the mean depression score was at the commonly used screening threshold for the GDS-15 (≥ 5) [26, 28]. This finding should be interpreted cautiously and should not be understood as a diagnostic indicator at the group level. However, it remains consistent with prior research identifying depression as one of the most common emotional problems in residential care [44]. In contrast, the mean GAI score remained below the commonly used screening threshold (≥ 9) [31], suggesting a more limited role of residential status in anxiety symptoms within this sample. Anger (STAXI) levels were moderate and somewhat higher than in the non-institutionalized group, which is consistent with the possibility that frustration and loss of control may be relevant emotional experiences in institutional settings [7]. Finally, loneliness reached values close to the high threshold on the UCLA-10 (≥ 20) [36, 45]. Loneliness scores were somewhat higher among institutionalized participants, although the group difference did not reach statistical significance. Therefore, these results should be interpreted cautiously and understood as descriptive of this sample rather than as evidence that institutionalization is, by itself, associated with greater loneliness.

The absence of a statistically significant difference in loneliness between residential groups may indicate that loneliness in later life is not determined solely by whether a person lives in an institutional or community setting. Instead, it may depend more strongly on relational factors such as the quality and continuity of meaningful social ties, opportunities for participation, and the subjective experience of connectedness in everyday life [11].

The pattern observed for anxiety and anger also deserves consideration. In the case of anxiety, the absence of significant group differences may suggest that residential status alone is not sufficient to explain anxious symptomatology in later life, which may depend more strongly on individual coping resources, health concerns, or perceived security within the living environment. Regarding anger, scores were moderate overall and somewhat higher in the institutionalized group, although the effect size was small. This finding may reflect the relevance of anger as an emotional response to frustration or loss of control in later life, while also being consistent with the possibility that anger in older adults is often experienced or expressed in less direct ways.

Institutionalized participants showed higher depressive symptoms and a tendency toward higher anger and loneliness scores. However, these findings should be interpreted with caution. Residential context may be associated with emotional well-being in later life, but it may also reflect prior and cumulative vulnerabilities that were not directly assessed in the present study, such as physical health, functional dependence, bereavement, or reduced social support [16, 21, 46]. For this reason, the observed group differences cannot be attributed to residential context alone.

Community-dwelling older adults may benefit from continuity in daily routines, greater autonomy in decision-making, and the preservation of close support networks, all of which have been identified as potentially protective factors for emotional well-being in later life [47, 48]. Likewise, institutional settings are themselves heterogeneous, and their emotional impact may vary depending on the quality of care, opportunities for participation, and continuity of social relationships [14, 15]. Overall, the comparison between institutionalized and non-institutionalized older adults suggests that residential context may be relevant to mental health in later life, posing an important challenge for care systems in aging societies. In this sense, residential status is a relevant but not sufficient condition for understanding well-being if relational and community dynamics are not taken into account. Living in the community does not automatically guarantee greater well-being, as loneliness and emotional distress may also occur in the absence of meaningful social support [49].

Building on this, the second objective of the study was to examine the role of loneliness in the relationship between residence, age, and emotional variables. The analyses highlighted loneliness as the most consistent factor associated with emotional distress in later life across the three models, whereas age and residential status showed more limited associations. This pattern is consistent with previous literature highlighting loneliness as a key factor in depression, anxiety, and related forms of emotional distress in older adults [17, 22, 45]. Moreover, the proportion of explained variance reached by the models is comparable to that of previous studies incorporating loneliness as a relevant explanatory factor [50, 51], further supporting the relevance of these findings. However, the results did not support a statistically significant indirect effects through loneliness in the relationship between age or residential status and the emotional outcomes. Subjective experiences of disconnection and loss of social bonds may therefore be closely linked to emotional distress in older adults, underscoring the need for policies and programs aimed at strengthening social ties and fostering a sense of belonging.

These findings emphasize the close interplay between individual well-being and the broader structures of health and social care. The consistent association between loneliness and emotional distress underscores the need for integrated community-based strategies that combine medical, psychological, and social perspectives. Strengthening collaborative networks between residential facilities, primary health care, and local community resources could enhance the capacity to prevent loneliness and mitigate its emotional consequences. In this regard, our results highlight the importance of moving beyond a purely biomedical model of care toward a holistic approach that prioritizes social connectedness and emotional well-being in later life.

This study is not without limitations. First, its cross-sectional design precludes firm conclusions about directionality or causality. Although the models were tested from an explanatory perspective, the findings should not be interpreted as demonstrating causal pathways or confirming mediation effects among residential status, loneliness, and emotional symptoms. Second, the comparability of the institutionalized and non-institutionalized groups was limited. Although age was statistically controlled in the main analyses, this adjustment should not be understood as fully compensating for broader structural differences between the groups. The groups may have differed in other relevant respects that were not measured or adjusted for, such as physical health, functional dependence, bereavement, socioeconomic conditions, and the quality and availability of social support. In this sense, institutionalization may reflect prior and cumulative vulnerability rather than residential context alone. Therefore, the observed associations should not be interpreted as attributable to institutional living per se. Third, the recruitment strategy was mixed and non-probabilistic, combining institutional collaboration with snowball sampling. This may have introduced selection bias, particularly in the community-dwelling group, and may limit the representativeness of the sample. Fourth, other potentially relevant variables were not included and could help explain the observed associations more precisely, such as frequency and quality of contact with relatives, continuity of external relationships, social participation, attachment-related variables, or perceived meaning and belonging. These factors may be especially important for understanding loneliness beyond residential status alone. Finally, all measures relied on self-report instruments, which may be affected by social desirability or recall bias. In addition, questionnaires were administered through different modes (in person and online depending on the context), which may also have influenced responses. Future research would benefit from a more differentiated assessment of loneliness in later life, particularly in institutional settings, in order to better capture the diversity of relational experiences and forms of social disconnection.

## Conclusions

This study highlights loneliness as a key factor associated with emotional distress in older adults. Although some differences were observed according to residential status, the models did not indicate statistically significant indirect effects through loneliness linking residential status or age to the emotional outcomes examined. These findings reinforce the importance of addressing loneliness and promoting meaningful social connectedness as relevant targets for psychosocial intervention in later life.

## Data Availability

The data that support the findings of this study are available from the corresponding author upon reasonable request.
